# A systematic review and meta-analysis of the effectiveness of hypertension interventions in faith-based organisation settings

**DOI:** 10.7189/jogh.13.04075

**Published:** 2023-10-13

**Authors:** Kit Yee Chan, Noori Srivastava, Zhicheng Wang, Xiaoqian Xia, Zhangziyue Huang, Adrienne N Poon, Daniel D Reidpath

**Affiliations:** 1 Centre for Global Health, Usher Institute, University of Edinburgh, Edinburgh, UK; 2 School of Social Sciences, Monash University, Clayton, Victoria, Australia; 3 Department of Medicine, School of Medicine & Health Sciences, George Washington University, Washington DC, USA; 4 China Development Research Foundation, Beijing, China; 5 London School of Hygiene & Tropical Medicine, London, UK; 6 Milken Institute School of Public Health, George Washington University, Washington DC, USA; 7 Institute for Global Health and Development, Queen Margaret University, Edinburgh, UK

## Abstract

**Background:**

Hypertension is the global, leading cause of mortality and is the main risk factor for cardiovascular disease. Community-based partnerships can provide cost-saving ways of delivering effective blood pressure (BP) interventions to people in resource-poor settings. Faith-based organisations (FBOs) prove important potential health partners, given their reach and community standing. This potential is especially strong in hard-to-reach, socio-economically marginalised communities. This systematic review explores the state of the evidence of FBO-based interventions on BP management, with a focus on randomised controlled trials (RCTs) and cluster RCTs (C-RCTs).

**Methods:**

Seven academic databases (English = 5, Chinese = 2) and grey literature were searched for C-/RCTs of community-based interventions in FBO settings. Only studies with pre- and post-intervention BP measures were kept for analysis. Random effects models were developed using restricted maximum likelihood estimation (REML) to estimate the population average mean change and 95% confidence interval (CI) of both systolic and diastolic blood pressure (SBP and DBP). The overall heterogeneity was assessed by successively adding studies and recording changes in heterogeneity. Prediction intervals were generated to capture the spread of the pooled effect across study settings.

**Results:**

Of the 19 055 titles identified, only 11 studies of fair to good quality were kept for meta-analysis. Non-significant, average mean differences between baseline and follow-up for the intervention and control groups were found for both SBP (0.78 mm of mercury (mmHg) (95% CI = 2.11-0.55)) and DBP (-0.20 mm Hg (95% CI = -1.16 to 0.75)). Subgroup analysis revealed a significant reduction in SBP of -6.23 mm Hg (95% CI = -11.21 to -1.25) for populations with mean baseline SBP of ≥140 mm Hg.

**Conclusions:**

The results support the potential of FBO-based interventions in lowering SBP in clinically hypertensive populations. However, the limited evidence was concentrated primarily in Christian communities in the US More research is needed to understand the implications of such interventions in producing clinically meaningful long-term effects in a variety of settings. Further research can illuminate factors that affect success and potential expansion to sites outside the US as well as non-Christian FBOs. Current evidence is inadequate to evaluate the potential of FBO-based interventions in preventing hypertension in non-hypertensive populations. Intervention effects in non-hypertensive population might be better reflected through intermediate outcomes.

Hypertension or high blood pressure (BP) is the global, leading cause of mortality and the main modifiable risk factor for cardiovascular disease [1-3], dementia [4], decline in kidney function [5] and severe illness and death from coronavirus disease 2019 (COVID-19) [6-8]. All these outcomes are associated with substantial health and economic costs as well as personal suffering. Hypertension can be prevented or managed through a series of interrelated strategies: (i) effective population measures that reduce population risk factors, (ii) hypertension awareness raising, (iii) screening and proper diagnosis, (iv) individual behaviour modification (e.g. diet, physical activity and tobacco use) and (v) improved access and adherence to medication [9,10]. Unfortunately, these strategies have not, at scale, successfully reached socio-economically marginalised populations [11-13]. Access barriers include (i) limited health budgets for NCD services that severely limit service availability, (ii) shortages of drugs and human resources (especially physicians) and (iii) a pervasive distrust of the health care system [14-16]. Finding low-cost strategies to improve service provision has become even more critical, considering the rising global noncommunicable disease (NCD) burden and the rapidly rising global economic pressure that further threatens the already lean health service budgets.

One effective, cost-saving strategy, is to shift certain primary and secondary hypertension prevention tasks (e.g. blood pressure screening, monitoring, health education, behavioural coaching) from physicians to allied health professionals (e.g. nurses, pharmacists) and/or trained members of the community [17-19]. Although various task-shifting models have emerged in the literature, much of the innovation relies on strategies for reaching diverse, underserved populations at locations where there is established trust between service providers and the populations [11,20]. The use of barbershops for hypertension intervention among African American males, in the US, is a case in point [21]. Other community sites for hypertension intervention include pharmacies [22] and community centres [23,24].

The potential of task-shifting has been illustrated in several recent systematic reviews and meta-analyses [18,25,26]. These reviews have shown significant mean BP reduction, in low-resource settings. With an estimated 84% of the world population being religiously affiliated [27] and with the long association of faith-based organisations (FBOs) with health care provision [28], FBOs stand out as potentially important partners for community-based BP intervention. In addition to their presence in some of the most socio-economically disadvantaged communities and their established trust within those communities, FBOs have resource advantages that may complement limited health sector resources [18,29]. These advantages include additional modes of financing and additional assets, such as human capital assets of paid staff and volunteers. Recent studies have shown the potential benefits of adapting community-based hypertension interventions to FBO settings [30,31]. However, the strength of the evidence has not been evaluated in aggregate, such as with meta-analyses [18,25,26].

Understanding the multi-faceted, complex factors and challenges or lessons-learned from FBO-based interventions would support development and evolution of models to guide future research and implementation of these interventions. This is best done using a systematic review of the literature. Thus, this systematic review aims to explore the state of the evidence of FBO-based interventions on BP management with a focus on randomised controlled trials (RCTs) and cluster randomised controlled trials (C-RCTs). In assessing the state of the literature, attention will be paid to the geographic spread of the studies, the nature of FBO involvement in the studies, and the factors that might have affected intervention success.

## METHODS

The review was guided by the Preferred Reporting Items for Systematic Reviews and Meta-Analyses (PRISMA) framework (Materials 1 in the **Online Supplementary Document**).

### Databases and search strategies

We conducted an extended search of the published literature, in English and Chinese using PubMed, EMBASE, PsycINFO, CINAHL, CNKI, SinoMed, and Google Scholar (first 100 hits). We focused on these seven English and Chinese academic databases due to resource constraints and the fact that they cover the vast majority of health sciences publications. Grey literature was also searched using The World Bank DataBank research paper repository and Google (first 100 titles). Searches were conducted using various combinations of the following search terms: “faith”/“region”-based, “hypertension”, “blood pressure”, “diabetes”, “cardiometabolic”/“cardiovascular” disease, “prevention”, “intervention”, “program”, “screening”, “education”, “health promotion”. Search criteria did not restrict publication date or language. Searches were conducted initially in July 2021 and updated for English and Chinese databases in July 2022 and June 2022, respectively. Materials 2 in the **Online Supplementary Document** details the search strategies for each database. English academic databases were independently searched by two reviewers (NS and ZH), while the Chinese databases were searched by ZCW.

### Inclusion and exclusion criteria

Included publications contain all of the following: (i) a NCD intervention that reported baseline and post-intervention BP measures (e.g. health promotion, risk factor screening, prevention, coaching, counselling to health promotion intervention/programmes), (ii) an intervention delivered in FBO settings and/or by trained FBO workers (e.g. FBO-based nurses, lay volunteers), (iii) cluster or individual randomised control trial (C-/RCT) design; and (iv) published in any languages. Excluded studies had the following characteristics: (i) were not original research (e.g. viewpoints, conference abstracts, reviews), (ii) were not trial studies, (iii) did not contain blood pressure as an outcome measure, or (iv) contained interventions that were purely based on faith-related practices (e.g. rituals, prayers).

### Quality assessment

Study quality was assessed for potential bias from randomisation, blinding, and outcome assessment using the NIH Study Quality Assessment Tool for Controlled Intervention Studies [32].

### Data extraction

Two reviewers independently extracted data from the retained studies (NS and KYC). Extracted data included first author, publication year, setting, religion, study population, objectives and design, sample size, follow-up time, key intervention components, intervention duration and intensity, training and roles assigned to FBO staff, and BP measures at baseline and follow-up. Where BP outcome data were available for two follow-up periods, the longer follow-up period was used. Task-shifting to FBO workers was categorised into one of three levels, using the team-based care conceptual framework adapted from Ogungbe et al. [18]. The categories were: (i) administrative tasks, (ii) basic intervention tasks (e.g. taking BP measures, health promotion, education, counselling), and (iii) advanced intervention tasks (diagnosis, treatment initiation and titration). Disagreements about extracted data were moderated by ZC and XQ.

### Data analysis

Meta-analysis of the pooled data was conducted from the eleven retained, randomised controlled trials using STATA version 16.0. Net changes in systolic blood pressure (SBP) and diastolic blood pressure (DBP) for the intervention and control groups were calculated separately based on the differences between the mean, respective measures at baseline and follow-up, and the standard deviation (SD). In nine of the 11 retained studies for SBP, and eight of the 10 retained studies for DBP, standard deviations were calculated from the reported confidence intervals and standard errors using the formulae outlined in Higgins et al. [33]. In the two remaining studies where this information was not reported [34,35], we imputed the values based on the mean standard deviation of the other eight (DBP) and nine (SBP) studies.

We estimated the effective sample size of each study to take into account the intra-class correlation (ICC) and design effect, as outlined in Killip et al. [36]. With one exception [37], ICCs were not reported. For the studies that did not report ICCs, the base cases were given fixed ICCs of 0.05. A sensitivity analysis was also conducted using lower and upper bounds of 0.02 and 0.09, respectively. These three values were based on reported ICCs in the literature for blood pressure studies in community settings. Studies were weighted using the inverse variance method. Study heterogeneity was quantified using *I*^2^ and Q statistics. A series of random effects models, using restricted maximum likelihood estimation (REML), were developed to estimate the population average mean change and 95% CI of both SBP and DBP. The significance of findings was set at *P* < 0.05. To assess each study's contribution towards the overall heterogeneity, a sensitivity analysis was conducted by successively adding studies and recording changes in heterogeneity. Prediction intervals were generated to capture the spread of the pooled effect across study settings.

Because the studies’ populations reflected different levels of clinical severity in hypertension, subgroup analyses were conducted. Studies were categorised into three subgroups based on the mean baseline BP: (i)>140 / 90 mm Hg; (ii) 130-139 / 80-89 mm Hg; (iii)<130 / 80 mm Hg. The subgroup corresponds to the categories of stage two hypertension and above, stage one hypertension, and alleviated blood pressure and below the ACC/AHA/AAPA/ABC/ACPM/AGS/APhA/ASH/ASPC/NMA/PCNA Guideline for the Prevention, Detection, Evaluation, and Management of High Blood Pressure in Adults [10]. Individual study effects and pooled effects were visualised with forest plots. Publication bias was assessed graphically through funnel plot asymmetry and statistically via Egger's regression test.

## RESULTS

### Study characteristics and the state of the evidence

**Figure 1:**
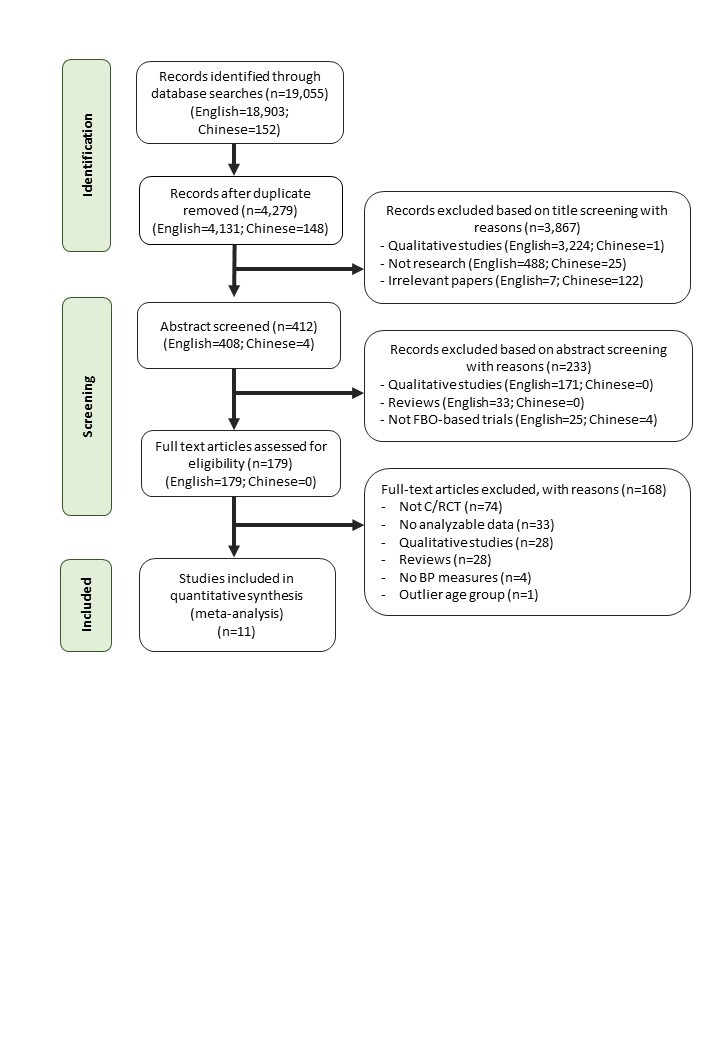
PRISMA flowchart showing search results and article selection. BP – blood pressure, C/RCT – cluster/randomised control trial

The searches yielded a total of 19 055 titles. After discarding duplicates, 4279 titles were screened. 3867 titles and 233 abstracts were excluded for failure to meet the inclusion criteria. Of the 179 full texts screened, 168 were excluded (**Figure 1**). Seventeen studies were kept as candidates for meta-analysis. Of these, four did not contain analysable data. Two studies were re-categorised as pre- and post-intervention design studies and discarded because identical BP interventions were provided to both the intervention and control groups while the experimental group received an additional faith component (e.g. prayer, gospel music). Noticeably, all studies that met the eligibility criteria were published in English. The inclusion of databases in the Chinese language did not yield additional studies. Our quality assessment indicates that the 11 retained studies are of fair to good quality (Materials 3 in the **Online Supplementary Document**).

**Table 1** summarises the key characteristics of the 11 studies kept for meta-analysis. All 11 studies were published in the last two decades (nine since 2010). The median sample size was 373 participants (range = 71-1257; interquartile range (IQR) = 660). The median intervention and follow-up duration were eight months (range = 1.5-15 months; IQR = 6.5 months) and 12 months (range = 4.5-15 months; IQR = 6 months), respectively. Despite the relatively large sample sizes of some of the studies, most contain interventions that had not been previously implemented in FBO settings. The recency of the publications and the pilot nature of the intervention together indicates the infancy of the evidence base for FBO-based BP interventions. Only two studies employed an individual RCT design, while the majority were C-RCTs, where randomisation occurred at the level of FBO. In one C-RCT [38], FBOs were first grouped into geographical regions prior to randomisation.

**Table 1:** Summary of the characteristics of the cluster-/randomised controlled trials included in the meta-analysis

Reference	Location	Religion	Study Population	Study Design	Sample size, individual (FBO)		Intervention duration (month)		Follow-up (month)	Task-shifting level*	Outcome measures		
					**Intervention group**	**Control group**					**Primary**	**Secondary**	
Tucker et al. (2019)	Florida, USA	Christianity (Methodist Episcopal)	African Americans	C-RCT	172 (11)	149 (10)	1.5		4.5	2	Nutrition label literacy, dietary intake, BP†, PA, weight	Nutrition label literacy, dietary intake, BP†, PA, weight	
Pengpid et al. (2019)	Nakhon Pathom, Thailand	Buddhism	Pre-hypertensive (120-139 / 80–89 mm Hg) and pre-diabetes (100-125 mg / dl)	C-RCT	220 (6)	223 (6)	6		6,12	0	SBP, BG		BMI, DBP, HDL, LDL, TC, TG, WC	
Schoenthaler et al. (2018)	New York, USA	Christianity (various denominations)	Hypertensive African Americans	C-RCT	172 (16)	201 (16)	5.5 (intensive = 2.5; maintenance = 3)		9,12	2	BP, MAP at 6th month		BP at 9th month	
Newton et al. (2018)	Louisiana, USA	Christianity (various denominations)	Pre-diabetic, diabetic African Americans	C-RCT	68 (4)	29 (4)		6	6	2	Weight		Body fat, BP, BG, cholesterol, quality of life, PA, food intake	
Brown et al. (2015)	Texas, USA	Christianity (Catholicism)	Hispanics and Non-Hispanic White adults	C-RCT	411 (5)	349 (5)		12	12	2	Fruit, vegetable and sodium intake, PA		BP	
Baig et al. (2015)	Illinois, USA	Christianity (Catholicism)	Diabetic Latinos	RCT	50	50		2	3,6	2	A1C		LDL, BP, weight, and diabetes self-care practices	
Wilcox et al. (2013)	South Carolina, USA	Christianity (Methodist Episcopal)	African Americans	C-RCT	749 (38)	508 (36)		15	15	2	BP, PA, fruit and vegetable intake		Fat- and fibre-related behaviours	
Duru et al. (2010)	Los Angeles, USA	Christianity (various denominations)	Physically inactive older female African Americans	RCT	37	34		8 (intensive = 2; maintenance = 6)	8	0	PA (steps walked)		PA (hours), BP, weight, pain score	
Yanek et al. (2001)	Baltimore, USA	Christianity (various denominations)	African American women with hypertension, diabetes mellitus or congestive heart failure	C-RCT	455 (9)	74 (7)		12	12	2	Weight, BMI, Body fat, BP, LDL HDL, energy intake, total fat, energy from fat, sodium, energy expenditure	Weight, BMI, Body fat, BP, LDL HDL, energy intake, total fat, energy from fat, sodium, energy expenditure	
Samuel-Hodge et al. (2009)	Central North Carolina, USA	Christianity (various denominations)	African Americans	C-RCT	117 (11)	84 (11)		8, 12	8, 12	2	A1C		Dietary intake, PA, BP, weight, diabetes knowledge, health status	
Paskett et al. (2018)	Appalachian counties, USA	Christianity	Patrons in 10 socio-economically disadvantaged regions	C-RCT	525 (13)	344 (15)		12	12	2	Weight		SBP, WHR, PA, fruit and vegetable intake	

BP – blood pressure, SBP – systolic blood pressure, DBP – diastolic blood pressure, A1C – glycosylated haemoglobin, BG – blood glucose, BMI – body mass index, HDL – high-density lipoprotein, LDL – low-density lipoprotein, MAP – mean arterial pressure, PA – physical activity, TC – total cholesterol, TG – triglyceride, WC – waist circumference, WHR – waist-to-hip ratio

*Task-shifting levels: 1. administrative tasks (e.g. participant recruitment); 2. simple intervention that can be performed independently with limited training and/or supervision support (e.g. health education, taking blood measures); 3. intervention tasks that require extended clinical training, supervision/support (e.g. diagnosis, prescription).

†Blood Pressure is inclusive of SBP and DBP.

Ten of the 11 studies were conducted in the US and were designed as community outreach interventions for high-risk, underserved, socioeconomically marginalised populations. All ten studies were based on FBOs of different Christian denominations. Nine studies targeted ethnic minorities (African American = 9, Hispanic/Latino = 2). Only one study was conducted outside the US in a non-Christian setting [39]. Intervention goals varied considerably between studies. Three studies targeted participants with one or more NCDs (hypertension [40], diabetes [38], diabetes mellitus, or congestive heart failure [41]). One study targeted pre-hypertensive populations [39]. The remaining seven studies targeted the more general patron populations and/or patrons with specific risk factors (e.g. physically inactive patrons). Noticeably, only two studies had baseline BP cut-offs in their participant selection criteria [39,40]. Furthermore, BP reduction was a primary outcome in less than half of the studies, amongst other more intermediate outcomes such as weight loss, increase in physical activity and dietary changes. However, heterogeneity in the selection of outcome measures and reporting means has ruled out meta-analysis based on risk factors.

Studies varied considerably in terms of intervention durations (median (mdn) = 6 months; range = 1.5-12 months) and follow-up time (mdn = 6 months; range = 4.5-15 months). Two studies had an intensive phase and a maintenance phase [35,40].

Intervention components and intensity also varied considerably between studies (**Table 1**). Most interventions also had multiple components delivered through two or more methods that ranged from individual and/or group sessions, telephone sessions, dedicated websites, and SMS reminders to print materials. The common component that appeared across all 11 studies was healthy behaviour or lifestyle education, counselling or coaching aimed at reducing NCD risks. However, the risk factor(s) of focus varied between studies. Some targeted multiple risk factors, while others a single risk factor. Some interventions also had components dedicated to the improvement of health advocacy and self-management of the targeted disease. Some interventions had a more hands-on approach and incorporated health-promoting activities into structured group sessions, e.g. physical exercise [35,40], healthy recipe tasting [42]. Others relied on a more self-directed and peer-supported approach [43].

Most of the interventions (n = 10) targeted individual patrons, though some studies also actively encouraged changes at the organisational level to support individual behavioural changes. For instance, the promotion of healthy food to be served at FBO-based events [37,38,44]) and/or the planning of physical activities by FBO staff, e.g. walking groups [38]. The outlier is Wilcox et al. [37], which focused solely on intervention at the organisational level – educating delegates to deliver healthy meals at the FBOs. This outlier also had the largest number of participants (n = 1257).

Most studies (n = 9) assigned some basic intervention tasks to trained FBO staff (e.g. health education, coaching, counselling, BP measures; i.e. level 2 tasks under the team-based care conceptual framework). However, the extent of task-shifting and the specific tasks designated to FBO workers varied between studies. The level of support offered to FBO staff also varied. Several of these studies involved FBO staff in the development and adaptation of the interventions [38,44]. Two studies gave no indications of involving FBO members in the development and delivery of the interventions [35,39]. However, in one of the two studies, FBOs workers assisted in participant recruitment [35]. Information on the adequacy of training and support was not available in most papers.

### Meta-analysis

**Figure 2:**
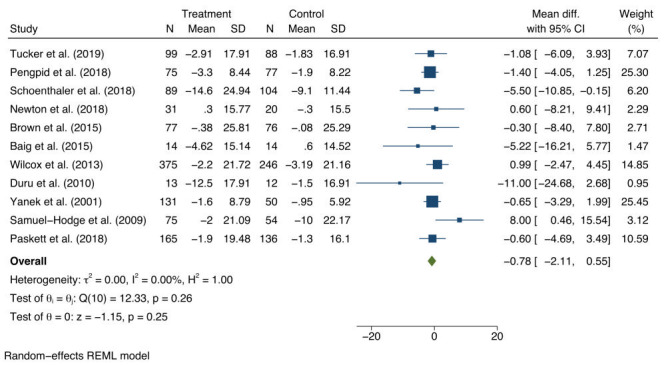
Estimates of the population average mean change and 95% confidence interval (CI) of systolic blood pressure (SBP) between intervention and control groups.

For SBP, a non-significant average mean difference was found of -0.78 mm Hg (95% CI = -2.11 to 0.55), between baseline and follow-up for the intervention and control groups (**Figure 2**). The 95% prediction interval for the SBP pooled estimate was -2.32 to 0.76. There was no measurable study heterogeneity. Asymmetry was not detected in the funnel plots (Materials 4a in the **Online Supplementary Document**). In addition, Egger’s regression test did not indicate evidence of publication bias (*t* = 1.63; degrees of freedom (df) = 11; *P* = 0.139).

**Figure 3:**
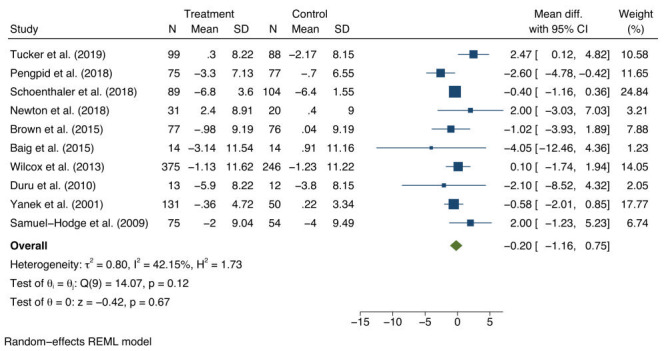
Estimates of the population average mean change and 95% confidence interval (CI) of diastolic blood pressure (DBP) between intervention and control groups.

For DBP, a non-significant average mean difference was found of -0.20 mm Hg (95% CI = -1.16 to 0.75), between baseline and follow-up for the intervention and control groups (**Figure 3**). The 95% prediction interval for the DBP pooled estimate was -2.56 to 2.15. There was non-significant study heterogeneity (*I*^2^ = 42.2%; τ^2^ = 0.8; Q-statistic (Q) (9) = 14.07; *P* = 0.12). Asymmetry was not detected in the funnel plots (Materials 4b in the **Online Supplementary Document**). In addition, Egger’s regression test did not indicate evidence of publication bias (*t* = 0.71; df = 11; *P* = 0.495).

**Figure 4:**
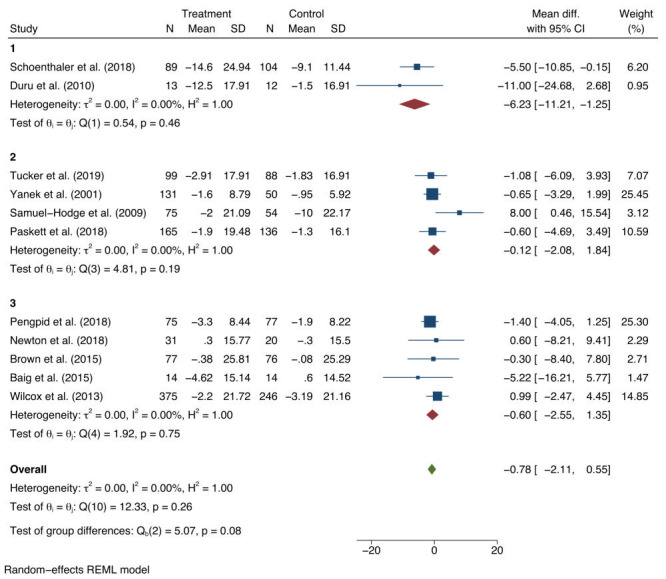
Subgroup analyses based on baseline population mean systolic blood pressure of: (1)>140 mm Hg; (2) 130-139 mm Hg; (3)<130 mm Hg. mmHg – millimetres of mercury

**Figure 5:**
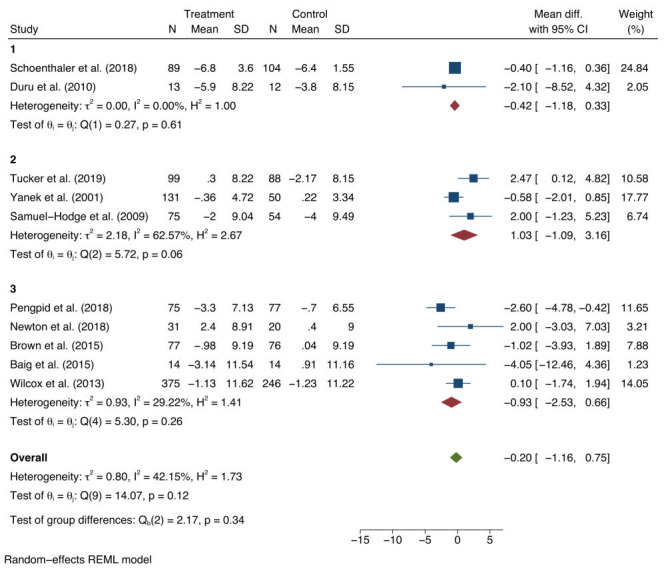
Subgroup analyses based on baseline population mean diastolic blood pressure of: (1)>90 mm Hg; (2) 80-89 mm Hg; (3) 80 mm Hg. mmHg – millimetres of mercury

Subgroup analyses of the SBP and DBP results were conducted. For SBP, the overall intervention effect was largely attributable to studies where the mean baseline SBP was greater than 140 mm Hg (-6.23 mm Hg; 95% CI = -11.21 to -1.25; *I*^2^ = 0.0%; *P* = 0.46) (**Figure 4**). Very little reduction in SBP was observed for studies where the mean SBP was less than 140 mgHg (-0.12 mm Hg; 95% CI = -2.08 to 1.84; *I*^2^ = 0.0%; *P* = 0.19), for studies with a mean baseline SBP measure of 130-139 mm Hg, (-0.60 mm Hg; 95% CI = -2.55 to 1.35; *I*^2^ = 0.0%; *P* = 0.75), and for studies with a mean baseline SBP measures under 130 mm Hg. No significant intervention effect was observed in any of the DBP subgroups (**Figure 5**).

## DISCUSSION

To our knowledge, this is the first systematic review and meta-analysis of the effect of interventions based in FBO settings on BP reduction. Our study did not find significant overall decreases in SBP or DBP levels. Although, a significant reduction in SBP was found in a subset of studies with a mean baseline population SBP level of ≥140 mm Hg (i.e. in those with clinical hypertension). Despite the non-significant overall effects, the direction of the findings is consistent with recent systematic reviews and meta-analyses on the effects of community-based interventions beyond FBO settings [18,25,26]. The weaker effect on BP reduction may be attributable to the lower mean baseline BP of the studies included in this current analysis, compared to previous meta-analyses. Previous studies have suggested that, especially with shorter-term interventions, intervention effects are often unstable for participants with baseline BP at the lower end of the spectrum [31]. Whilst only 18% of studies in the current review had a mean population SBP level of ≥140 mm Hg at baseline, 65% of the CHW-led interventions in the review by Anand et al. had a baseline population mean SBP of ≥140 mm Hg [25]. The proportion of studies with baseline population mean SBP level in the lower end of the BP spectrum was also much higher in our study (≥130 mm Hg = 36%; 120-129 mm Hg = 40%; >120 mm Hg = 5%), compared to the studies included in the aforementioned review (130-139 mm Hg = 13%; 120-129 mm Hg = 20%; <120 mm Hg = 2%).

A potential contributor to the low average population baseline BP of the studies retained in our review could be that most of these studies were primarily aimed at reducing risk factors in the general adult patrons rather than BP reduction in hypertensive patrons. Given the cumulative nature of NCD risks over the life course, the value of organisation-based interventions capable of altering members’ risk trajectories prior to the emergence of the disease cannot be understated. To enable a more informative meta-analysis of BP outcomes from such interventions, breakdowns of BP outcomes by participants’ baseline BP ranges would be required but are absent in the publications used in this review. Moreover, evaluations of interventions that target non-hypertensive patrons should not rely on BP outcomes alone but in conjunction with intermediate outcome measures, such as changes in risk factors (e.g. weights, dietary changes), disease knowledge, and self-advocacy in health-seeking. Unfortunately, even though most of the studies we identified had included such measures, heterogeneity in the selection and reporting of outcome measures prevented further analyses. For a more complete understanding of the potential of FBO-based intervention on NCD prevention, separate reviews on the effects on some of the more commonly used intermediate outcomes will be required.

Consistent with previous reviews, the tasks delegated to lay-FBO workers were mostly basic intervention tasks that did not require substantial training (e.g. health education, counselling, BP measures). Previous research has shown that while interventions assisted or led by trained community workers can lead to effective BP reduction, their effectiveness might be inferior to interventions led by allied health workers (e.g. dietitians, pharmacists) [18,25]. Most studies identified in our review have multiple components and involved a mix of trained FBO workers and allied health workers in the delivery of interventions. Further research will be needed to provide insights into which intervention components might be best delivered by trained FBO workers vs. allied health workers. To be truly effective, community-based interventions must be integrated within the wider health system, allowing participants with high BP to have easy access to medical consultation for diagnosis and prescriptions [17,18,25]. Unfortunately, most studies identified in our review appeared to be standalone interventions with no mentions of wider health system linkages – an issue that perhaps requires consideration in future studies.

Several other limitations of the evidence base and methodological issues require further elaboration. First, despite the comprehensiveness of our search strategies and databases, only eleven publications were identified globally. Aside from one study conducted in Thai Buddhist temples, all the other studies were conducted in Christian FBOs in a single high-income country – the US. The absence of FBO-based studies from China is understandable, considering China already has a three-tier health care delivery system with a well-established history of incorporating lower-order health professionals and trained community health workers (formerly known as “barefoot doctors”) into health promotion and disease prevention strategies [45]. The challenge for China has largely been the strengthening and transitioning of the already established system to NCD prevention and management, which has been a focus of recent NCD intervention strategies and intervention research [46-48]. Furthermore, the insignificant roles of organised religions in the daily lives of ordinary Han Chinese (who make up 91.1% of the Chinese population) [49] also make FBOs less suitable for community-based NCD interventions. The potential for FBOs as partners in health promotion might be of higher value in ethnic minority populations, where organised religions have a greater presence.

More surprising, however, is the absence of studies from countries, besides the US, in which organised religions have much greater prominence (e.g. South Asian, African and Middle Eastern countries). The omission of academic databases in languages besides English and Chinese (e.g. LILACS) from the current review could have led to the oversight of studies published in other languages. However, what is evident is the lack of research in even English-speaking countries where there is a prominent FBO presence, such as in sub-Saharan Africa. Given the reach and resources available to the FBO sector and health budget shortages, especially in low- and middle-income countries (LMICs), the lack of health-religion cooperation in health research and service delivery could represent a missed opportunity for health outcome improvement [50].

Relative to previous reviews, the samples sizes of the studies identified in our review were considerably smaller – 30% (range = 71-1257) had ≥750 participants (range = 118-2397) compared to, for example, 90% of the CHW-led studies in Anand et al. [25]. Intervention duration and follow-up intervals were on average shorter in the studies used in our meta-analysis than those reported in previous reviews. While our quality assessment indicates the studies in our analyses to be of fair to good standards, the strength of our analysis was negatively affected by potential bias resulting from issues including unclear treatment allocation, concealment, drop-out rates and protocol adherence, and insufficient analysis and reporting in C-RCTs.

As mentioned earlier, the studies identified by our review varied considerably in intervention components, intensity, duration, follow-up intervals as well as the involvement, training, and support for FBO workers. To better understand the potential of FBOs as partners for community-based hypertension interventions, more research will be needed across more FBO settings, especially in countries with strong FBO presence. More research will also be required on the longer-term effects of successful FBO-based interventions as well as how some of the aforementioned factors affect hypertension and other intermittent intervention outcomes. Finally, this review only included evidence at the highest level (i.e. C/RCT). The inclusion of studies with other designs (e.g. quasi-experimental design) might have interesting insights into FBO-based intervention, or at the very least, provide additional data to supplement those identified in the current review.

## CONCLUSIONS

This review provided support for the potential of FBO-based intervention in reducing blood pressure in hypertensive populations in the short term. However, more research is needed to understand the effects of such interventions in producing clinically meaningful long-term effects and the factors that affect their success. The current evidence base is inadequate for evaluating the potential of FBO-based interventions on hypertension prevention in non-hypertensive populations because intervention effects might be better reflected through intermediate outcomes.

## Additional material


Online Supplementary Document
