# Supplementary material for: A systematic review and meta-analysis of the effectiveness of hypertension interventions in faith-based organisation settings
Source: J Glob Health. 2023 Oct 13;13:04075. doi: 10.7189/jogh.13.04075 (PMC10570758; doi:10.7189/jogh.13.04075)

ONLINE SUPPLEMENTARY DOCUMENT

Title: A systematic review and meta-analysis of the effectiveness of hypertension interventions in faith-based organisation settings

Kit Yee Chan, Noori Srivastava, Zhicheng Wang, Xiaoqian Xia, Zhangziyue Huang, Adrienne N Poon, Daniel D Reidpath

Supplementary Materials 1. PRISMA CheckList

Section and Topic	Item #	Checklist item	Location where item is reported
**TITLE**			
Title	1	Identify the report as a systematic review.	Title
**ABSTRACT**			
Abstract	2	See the PRISMA 2020 for Abstracts checklist.	Abstract
**INTRODUCTION**			
Rationale	3	Describe the rationale for the review in the context of existing knowledge.	Introduction
Objectives	4	Provide an explicit statement of the objective(s) or question(s) the review addresses.	Introduction
**METHODS**			
Eligibility criteria	5	Specify the inclusion and exclusion criteria for the review and how studies were grouped for the syntheses.	Methods
Information sources	6	Specify all databases, registers, websites, organisations, reference lists and other sources searched or consulted to identify studies. Specify the date when each source was last searched or consulted.	Methods
Search strategy	7	Present the full search strategies for all databases, registers and websites, including any filters and limits used.	Supplementary Materials
Selection process	8	Specify the methods used to decide whether a study met the inclusion criteria of the review, including how many reviewers screened each record and each report retrieved, whether they worked independently, and if applicable, details of automation tools used in the process.	Methods
Data collection process	9	Specify the methods used to collect data from reports, including how many reviewers collected data from each report, whether they worked independently, any processes for obtaining or confirming data from study investigators, and if applicable, details of automation tools used in the process.	Methods
Data items	10a	List and define all outcomes for which data were sought. Specify whether all results that were compatible with each outcome domain in each study were sought (e.g. for all measures, time points, analyses), and if not, the methods used to decide which results to collect.	Methods
	10b	List and define all other variables for which data were sought (e.g. participant and intervention characteristics, funding sources). Describe any assumptions made about any missing or unclear information.	Methods
Study risk of bias assessment	11	Specify the methods used to assess risk of bias in the included studies, including details of the tool(s) used, how many reviewers assessed each study and whether they worked independently, and if applicable, details of automation tools used in the process.	Methods
Effect measures	12	Specify for each outcome the effect measure(s) (e.g. risk ratio, mean difference) used in the synthesis or presentation of results.	Methods
Synthesis methods	13a	Describe the processes used to decide which studies were eligible for each synthesis (e.g. tabulating the study intervention characteristics and comparing against the planned groups for each synthesis (item #5)).	Methods
	13b	Describe any methods required to prepare the data for presentation or synthesis, such as handling of missing summary statistics, or data conversions.	Methods

Section and Topic	Item #	Checklist item	Location where item is reported
	13c	Describe any methods used to tabulate or visually display results of individual studies and syntheses.	Methods
	13d	Describe any methods used to synthesize results and provide a rationale for the choice(s). If meta-analysis was performed, describe the model(s), method(s) to identify the presence and extent of statistical heterogeneity, and software package(s) used.	Methods
	13e	Describe any methods used to explore possible causes of heterogeneity among study results (e.g. subgroup analysis, meta-regression).	Methods
	13f	Describe any sensitivity analyses conducted to assess robustness of the synthesized results.	Methods
Reporting bias assessment	14	Describe any methods used to assess risk of bias due to missing results in a synthesis (arising from reporting biases).	Methods
Certainty assessment	15	Describe any methods used to assess certainty (or confidence) in the body of evidence for an outcome.	Methods
**RESULTS**			
Study selection	16a	Describe the results of the search and selection process, from the number of records identified in the search to the number of studies included in the review, ideally using a flow diagram.	Results
	16b	Cite studies that might appear to meet the inclusion criteria, but which were excluded, and explain why they were excluded.	Figure 1
Study characteristics	17	Cite each included study and present its characteristics.	Results
Risk of bias in studies	18	Present assessments of risk of bias for each included study.	Supplementary Materials
Results of individual studies	19	For all outcomes, present, for each study: (a) summary statistics for each group (where appropriate) and (b) an effect estimate and its precision (e.g. confidence/credible interval), ideally using structured tables or plots.	Results
Results of syntheses	20a	For each synthesis, briefly summarise the characteristics and risk of bias among contributing studies.	Results
	20b	Present results of all statistical syntheses conducted. If meta-analysis was done, present for each the summary estimate and its precision (e.g. confidence/credible interval) and measures of statistical heterogeneity. If comparing groups, describe the direction of the effect.	Results
	20c	Present results of all investigations of possible causes of heterogeneity among study results.	Results
	20d	Present results of all sensitivity analyses conducted to assess the robustness of the synthesized results.	Results
Reporting biases	21	Present assessments of risk of bias due to missing results (arising from reporting biases) for each synthesis assessed.	Results
Certainty of evidence	22	Present assessments of certainty (or confidence) in the body of evidence for each outcome assessed.	Results
**DISCUSSION**			
Discussion	23a	Provide a general interpretation of the results in the context of other evidence.	Discussion
	23b	Discuss any limitations of the evidence included in the review.	Discussion
	23c	Discuss any limitations of the review processes used.	Discussion
	23d	Discuss implications of the results for practice, policy, and future research.	Discussion

Section and Topic	Item #	Checklist item	Location where item is reported
**OTHER INFORMATION**			
Registration and protocol	24a	Provide registration information for the review, including register name and registration number, or state that the review was not registered.	Supplementary Materials*
	24b	Indicate where the review protocol can be accessed, or state that a protocol was not prepared.	Not Prepared
	24c	Describe and explain any amendments to information provided at registration or in the protocol.	Not Applicable
Support	25	Describe sources of financial or non-financial support for the review, and the role of the funders or sponsors in the review.	Funding
Competing interests	26	Declare any competing interests of review authors.	Competing Interests
Availability of data, code and other materials	27	Report which of the following are publicly available and where they can be found: template data collection forms; data extracted from included studies; data used for all analyses; analytic code; any other materials used in the review.	Not Applicable

*This review was not registered. From: Page MJ, McKenzie JE, Bossuyt PM, Boutron I, Hoffmann TC, Mulrow CD, et al. The PRISMA 2020 statement: an updated guideline for reporting systematic reviews. BMJ 2021;372:n71. doi: 10.1136/bmj.n7. For more information, visit: http://www.prisma-statement.org/

Supplementary Materials 2. Search strategies

Database	Search Syntax
**Pubmed**	(faith-based OR faithbased OR faith place OR religion-based OR religious-based OR church-based OR temple-based OR mosque-based OR shrine-based Or synagogue-based OR gurdwara-based) AND (hypertension or blood pressure or diabetes or cardiometabolic disease or cardiovascular disease) AND (prevention OR intervention OR program OR screening OR education OR health promotion)
**EMBASE**	(faith-based or faithbased or faith place or religion-based or religious-based or church-based or temple-based or mosque-based or shrine-based or synagogue-based or gurdwara-based).mp. [mp=title, abstract, heading word, drug trade name, original title, device manufacturer, drug manufacturer, device trade name, keyword, floating subheading word, candidate term word]<br><br>((faith-based or faithbased or faith place or religion-based or religious-based or church-based or temple-based or mosque-based or shrine-based or synagogue-based or gurdwara-based) and (hypertension or blood pressure or diabetes or cardiometabolic disease or cardiovascular disease)).mp. and (prevention or intervention or program or screening or education or health promotion).af. [mp=title, abstract, heading word, drug trade name, original title, device manufacturer, drug manufacturer, device trade name, keyword, floating subheading word, candidate term word]<br><br>faithbase.af.<br>faith-based.af.<br>faith place.af.<br>religion-based.af.<br>church-based.af.<br>mosque-based.af.<br>blood pressure.af.<br>blood pressure<br>cardiometabolic disease.af.<br>cardiometabolic disease
**CINHAL**	(faith-based OR faith place OR religion-based OR religious-based OR church-based OR temple-based OR mosque-based OR shrine-based Or synagogue-based OR gurdwara-based) AND (hypertension or blood pressure or diabetes or cardiometabolic disease or cardiovascular disease) AND (prevention OR intervention OR program OR screening OR education OR health promotion)

Database	Search Syntax
**PsycINFO**	faith-based.af.<br>faith place.af.<br>religion-based.af.<br>religious-based.af.<br>church-based.af.<br>temple-based.af.<br>mosque-based.af.<br>shrine-based.af.<br>synagogue-based.af.<br>gurdwara-based.af.<br>hypertension.af.<br>blood pressure.af.<br>diabetes.af.<br>cardiometabolic disease.af.<br>cardiovascular disease.af.<br>prevention.af.<br>intervention.af.<br>program.af.<br>screening.af.<br>education.af.<br>health promotion.af.<br>1 or 2 or 3 or 4 or 5 or 6 or 7 or 8 or 9 or 10<br>11 or 12 or 13 or 14 or 15<br>16 or 17 or 18 or 19 or 20 or 21<br>22 and 23 and 24
**Google Scholar**	To accommodate the character limit allowed in Google Scholar, our overall strategy was broken into three separate searches:<br><br>(faith-based OR faith place OR religion-based) AND (hypertension OR blood pressure OR diabetes or cardiometabolic disease OR cardiovascular disease) AND (prevention OR intervention OR program OR screening OR education OR health promotion)<br><br>(religious-based OR church-based OR temple-based) AND (hypertension OR blood pressure OR diabetes OR cardiometabolic disease OR cardiovascular disease) AND (prevention OR intervention OR program OR screening OR education OR health promotion)<br><br>(mosque OR shrine OR synagogue OR gurdwara) AND (hypertension OR blood pressure OR diabetes OR cardiometabolic disease OR cardiovascular disease) AND (prevention OR intervention OR program OR screening OR education OR health promotion)

Database	Search Syntax
**Google**	To accommodate the 32 words limit allowed in Google Searches, the search strategy was broken into two separate searches:<br><br>(faith-based OR faith place OR religion-based OR religious-based OR church-based OR temple-based OR mosque-based) AND (hypertension OR blood pressure OR diabetes OR cardiometabolic disease OR cardiovascular disease) AND (prevention OR intervention OR program OR screening OR education OR health promotion)<br>limit to first 100<br><br>(shrine-based OR synagogue-based OR gurdwara-based) AND (hypertension OR blood pressure OR diabetes OR cardiometabolic disease OR cardiovascular disease) AND (prevention OR intervention OR program OR screening OR education OR health promotion)<br><br>Based on the number of FBO-related search terms in the two search strategies, we had planned to scan through the first 70 titles in the results from the first strategy and the first 30 titles of the second strategy. However, the first strategy yields 120,000 results, while the second yields only 8. We had therefore reviewed the first 92 results from the first search strategy and all eight results from the second.
**SinoMed and CNKI**	#1 (宗教 OR 佛教 OR 道教 OR 伊斯兰教 OR 天主教 OR 基督教 OR 寺庙 OR 道观 OR 清真寺 OR 教堂 OR 古尔邦节)<br>#2 (血压 OR 糖尿病 OR 心血管疾病 OR 心脏病)<br>#3 (预防 OR 干预 OR 项目 OR 筛查 OR 教育 OR 健康促进)<br>#4 (#1 AND #2 AND #3)

Supplementary Materials 3. Study Quality Assessment using the NIH Quality Assessment of Controlled Intervention Studies

	Tucker et al (2109)	Pengpid et al (2019)	Schoenthaler et al (2018)	Newton et al. (2018)	Brown et al. (2015)	Baig et al (2015)	Wilcox et al (2013)	Duru et al (2010)	Yanek et al (2001)	Samuel-Hodge et al. (2009)	Paskett et al. (2018)
RCT/Cluster RCT	Yes	Yes	Yes	Yes	Yes	Yes	Yes	Yes	Yes	Yes	Yes
Adequate random-isation	Yes	Yes	Yes	Yes	Yes	Yes	Yes	Yes	Yes	Yes	Yes
Treatment allocation concealed	NR	Yes	Yes	Yes	NR	NR	NR	Yes	No	Yes	NR
Participants & providers blinded to group assignment	NR	NR	NR	No	No	NR	NR	No	NR	NR	NR
Assessors blinded to group assignment	NR	NR	NR	NR	No	NR	NR	Yes	NR	NR	NR
Groups similar at baseline	Yes	Yes	Yes	Yes	Yes	Yes	Yes	Yes	Yes	Yes	Yes
Overall drop-out rate ≤20%	Yes	Yes	No	No	Yes	No	No	Yes	No	Yes	No
≤15% differential drop-out rate between groups	No	Yes	Yes	No	Yes	Yes	Yes	Yes	No	Yes	Yes
High adherence to intervention protocols	No	CD	No	No	No	No	NR	No	No	No	No
Other interventions avoided	Yes	Yes	Yes	Yes	Yes	Yes	Yes	Yes	Yes	Yes	Yes
Valid & reliable outcome measures used	Yes	Yes	Yes	Yes	Yes	Yes	Yes	Yes	Yes	Yes	Yes
Sufficient sample size	No	Yes	Yes	No	No	NR	Yes	NR	NR	No	NR
Outcomes reported or subgroups analysis prespecified	Yes	Yes	Yes	Yes	Yes	Yes	Yes	Yes	Yes	Yes	Yes
Intention-to-Treat (ITT) analysis	No	No	Yes	Yes	Yes	Yes	Yes	Yes	Yes	No	Yes
**Overall Quality Rating**	**Fair**	**Good**	**Good**	**Fair**	**Good**	**Good**	**Good**	**Good**	**Fair**	**Fair**	**Fair**

Note: Randomised control trial (RCT), Cannot decide (CD), Not Reported (NR)

Supplementary Materials 4a: SBP Funnel plot

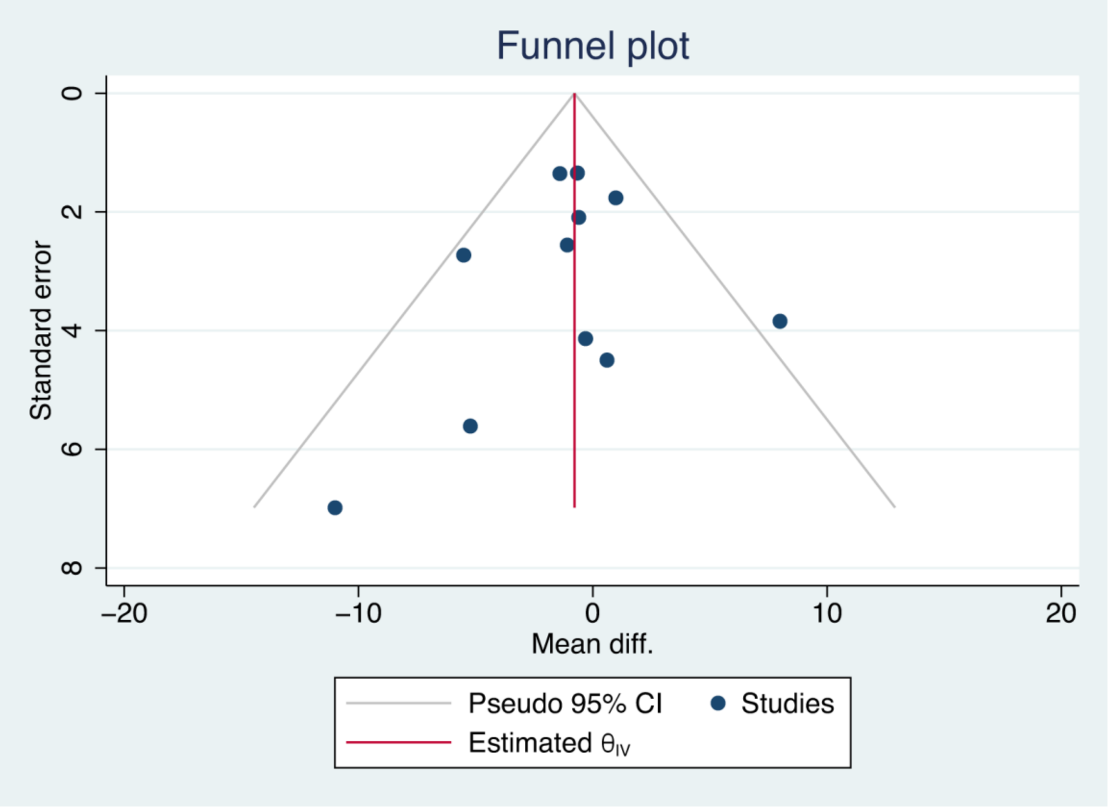

Supplementary Materials 4b. DBP Funnel plot

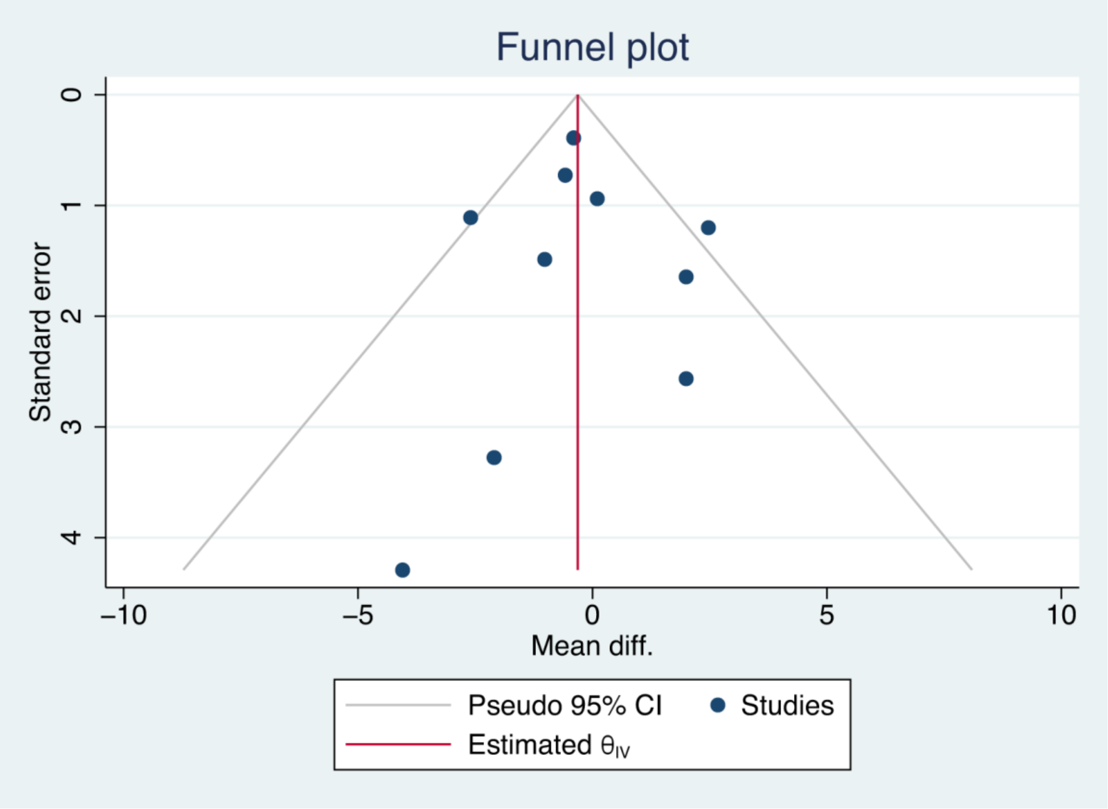

Supplement: Online Supplementary Document [file jogh-13-04075-s001.pdf]
